# Emotion Regulation Strategies, Workload Conditions, and Burnout in Healthcare Residents

**DOI:** 10.3390/ijerph17217816

**Published:** 2020-10-26

**Authors:** Ramón Martín-Brufau, Alejandro Martin-Gorgojo, Carlos Suso-Ribera, Eduardo Estrada, María-Eugenia Capriles-Ovalles, Santiago Romero-Brufau

**Affiliations:** 1 Psychiatry Department, Mental Health Center of Lorca, 30800 Lorca, Spain; ramonmail@gmail.com; 2 STI/Dermatology Department, Madrid City Council, 28006 Madrid, Spain; alejandromartingorgojo@aedv.es; 3 Department of Personality, Assessment and Clinical Psychology, Jaume I University, 12071 Castellón de la Plana, Spain; 4 Department of Social Psychology and Methodology, Universidad Autónoma de Madrid, 28049 Madrid, Spain; eduardo.estrada.rs@gmail.com; 5 Emergency Department, Clinical University Hospital of Valencia, 46010 Valencia, Spain; marucapriles@gmail.com; 6 Department of Medicine, Mayo Clinic, Rochester, Rochester, MN 55905, USA; santiagoromerobrufau@gmail.com; 7 Department of Biostatistics, Harvard T. H. Chan School of Public Health, Boston, MA 02115, USA

**Keywords:** emotion regulation, emotional suppression, cognitive reevaluation, burnout, residency

## Abstract

Background: Burnout syndrome is very prevalent among healthcare residents. Initiatives addressing workload conditions have had limited impact on burnout. The present study aims to explore the contribution of two emotion regulation strategies, namely emotion suppression and cognitive reevaluation, to residents’ burnout, while accounting for workload factors. Methods: Participants were 105 residents (68.6% women; mean age = 27.5, *SD* = 3.0). They completed measures of workload, burnout, and emotion regulation. The study was cross-sectional. Results: Emotional suppression was associated with higher burnout (depersonalization scale; *β* = 0.20, *p* < 0.05, CI 0.15–2.48) and cognitive revaluation was linked to lower burnout (higher personal accomplishment; *β* = 0.35, *p* < 0.01, CI 0.16–2.56), even after controlling for demographic and workload factors. We found interaction effects between workload variables (supervisor support and number of patient hours) and emotion regulation (*p* < 0.05). Conclusions: The relationship between workload, emotion regulation, and burnout seems to be complex. That is, similar work conditions might generate different levels of burnout depending on the resident’s emotional regulation strategies. This might partly explain why existing initiatives based on workload changes have had a modest impact on burnout. Results also support including emotion regulation training in prevention and treatment programs targeting burnout during residency.

## 1. Introduction

Burnout is a serious problem among healthcare professionals, such as nurses and physicians [1,2,3]. Burnout is associated with a number of deleterious consequential outcomes in the healthcare provider, such as an increased number of sick leave absences, job turnover, increased risk of psychopathology and physical complications, and poorer quality of healthcare delivery [4,5]. Moreover, this ineffective response to stress is likely to negatively influence patient outcomes (e.g., poor treatment compliance and less satisfaction with treatment). Furthermore, these potentially adverse outcomes can contribute to exerting additional strain on the healthcare professional, thus creating a vicious cycle [6].

The problem of burnout appears to be particularly alarming during residency, where between 36% and 76% of residents have experienced this syndrome anytime during residency [7,8]. Several factors have been proposed to influence burnout during residency, including gender, age, marital status, culture, workload, physical activity, and coping strategies, among others [9,10,11,12]. Of these, workload interventions have received the most attention. However, existing treatments have had a limited impact on burnout levels [1], and public time reduction initiatives have not solved the problem [13].

Both clinical and organizational studies have highlighted the importance of emotion regulation strategies on individual outcomes, including burnout in healthcare professionals [14,15,16,17]. However, their role in residents’ burnout, as well as their contribution above and beyond already explored variables like workload, remains unclear. The present study aims to provide evidence on the contribution of emotion regulation strategies to residents’ burnout while controlling for important covariates. Emotion regulation is a complex phenomenon influenced by an interconnected set of neuroendocrine, neurological, genetic, personality, and situational factors [18,19,20,21,22]. In this work, we focus on two important regulatory mechanisms embedded in this complex network of factors, namely emotional suppression and cognitive reevaluation [23].

Emotion regulation can be defined as “the process by which individuals influence which emotions they have, when they have them, and how they experience and express these emotions” [24]. Since Dr. Hochschild’s pioneering work [25], emotion regulation strategies have been frequently classified as surface (superficial) or deep. In surface acting, emotional expressions are adjusted to what is desired by others, but internal feelings are not necessarily modified. Therefore, a discrepancy between internally felt and required emotions usually emerges. By contrast, in deep acting, inner feelings are actively regulated and adjusted to what is displayed externally [26]. Emotional suppression and cognitive reevaluation are the most frequent methods of surface and deep acting, respectively. Emotional suppression is characterized by inhibiting true feelings in favor of desired emotions [27]. As opposed to this, individuals using cognitive reevaluation reappraise the situation so that this new evaluation leads to the required emotion [14,28]. In general, surface acting strategies, like emotional suppression, are associated with poorer outcomes, while deep acting tends to be a recommended practice [29,30].

An example of emotional suppression and cognitive reevaluation strategies is provided in the context of medical residents. In organizational settings, emotion regulation tends to be understood as how employees manage emotions to adapt to work demands [31]. Residents face high responsibility demands, deal with critical life or death situations, experience patient and family demanding pressures, and often have little time to prepare an elaborated response to daily challenges. The previous, together with the fact that residents tend to be young and generally lack professional experience, frequently leads to feelings of insecurity [32]. In this scenario, an emotional suppression strategy would be to try to hide such feelings by trying to be seen as confident by others at any cost or by using technical words in a patient’s presence, thus showing ability and knowledge [33]. In contrast, in this same scenario, residents may decide to remind themselves that they are still trainees who are expected to lack specific knowledge and abilities and use this to challenge themselves to improve, which is an example of a cognitive reevaluation strategy.

In the current study, we explore how these two regulation strategies, namely emotional suppression and cognitive reevaluation, are related to burnout among hospital residents and how much they contribute above and beyond previously investigated risk and protective factors, like workload and demographic variables, which will be used as covariates. In line with studies suggesting the need to explore factors that facilitate or hinder burnout processes [34], we will also examine whether the relationship between emotion regulation and burnout is contextually determined (i.e., moderated) by these covariates.

**Hypothesis** **1** **(H1).** *We expect that cognitive reevaluation will be associated with lower burnout levels when compared to emotional suppression, even after adjusting for demographic and workload variables.*

**Hypothesis** **2** **(H2).** *We also hypothesize that emotion regulation strategies and some covariates will interact—that is, that the contribution of emotion regulation strategies on burnout will depend on the context in which they occur, in line with previous clinical research [35].*

## 2. Materials and Methods

### 2.1. Participants

A description of participants’ characteristics is shown in Table 1. A total of 105 residents in the Clinico Hospital of Valencia, Spain (68.6% women; age range = 21–39 years; mean age = 27.5, *SD* = 3.0) participated in the study. The sample size was sufficient according to the recommendations of the required sample sizes for multiple regression analyses [36,37,38]. The survey was sent to 150 subjects (response rate was 70.0%). Residents in an external hospital at that moment or on vacation were excluded. The majority of residents participated in a clinical specialty program (83.0%). The fourth year of residency allows for a period of training in an external hospital, which explains the decrease in the number of residents in that year. Both nurses and physicians were asked to participate, given that burnout problems are frequent and similar in both professional roles [30].

### 2.2. Procedure

Medicine and nurse residents from all residency years were sent an online questionnaire with an invitation to participate. Two periods of data collection were planned to increase the number of participants. These were February 2013 and again in March 2014 (only non-respondents and new residents were selected for the second administration). The survey was available online for one month each time. When the two periods were finished, the information was downloaded and analyzed. All procedures followed were in accordance with the 1964 Helsinki declaration, the study protocol, and good clinical practice. Informed consent was obtained from all participants included in the study, and privacy was granted (no identifying information was obtained). There was no economic compensation for participation.

### 2.3. Instruments

#### 2.3.1. Maslach Burnout Inventory (MBI)

We used a validated version of the Maslach Burnout Inventory (MBI) in Spanish [39]. The MBI has 22 items that represent three dimensions of burnout syndrome: emotional exhaustion (9 items), depersonalization (5 items), and personal accomplishment (8 items). Emotional exhaustion evaluates feelings of being fatigued and emotionally overwhelmed by work. Depersonalization reflects the extent to which an individual responds impersonally and lacking sensitivity towards people whom they care for, provide service to, or instruct at work. Personal accomplishment is the perceived personal competence and achievement at work. A global burnout score can also be calculated by summing all subscale scores [40]. However, the use of the subscales is preferred [34]. The MBI is considered the “gold-standard” tool for measuring burnout and has become the most frequently and widely used instrument for its assessment [8,41]. We used the cut-off points used in other burnout studies to classify residents at risk for emotional exhaustion, depersonalization, and personal accomplishment [42]. These were >27, >10, and <33 for high emotional exhaustion, high depersonalization, and low personal accomplishment, respectively. Medium levels of burnout are represented by scores ranging from 19 to 26 (emotional exhaustion), 6 to 9 (depersonalization), and 34 to 39 (personal accomplishment). Ratings below 18 and 5 have been said to reflect low emotional exhaustion and depersonalization, respectively, and ratings above 40 are argued to indicate high personal accomplishment. This strategy is based on the MBI scores’ correspondence with levels of psychopathology and psychiatric diagnoses [43].

#### 2.3.2. Emotion Regulation Questionnaire (ERQ)

We used the ERQ to evaluate emotion regulation strategies [44]. This instrument has been previously adapted to Spanish and with good reliability and validity results [45]. The questionnaire has ten items that evaluate two regulatory strategies investigated in the present investigation: emotional suppression (4 items) and cognitive reevaluation (6 items). Each item has a 1-to-7 scale range, where 1 represents totally disagree, and 7 reflects totally agree.

#### 2.3.3. Demographic and Workload Information

As described before, burnout has been associated with demographic variables and work-related characteristics in some studies [9]. Therefore, in the online survey, we included demographic information, as well as job workload data and residents’ subjective work experience. A set of questions was created ad hoc in the online survey to assess the following variables (see Tables S1 and S2):

1. Demographic characteristics: sex, age, civil status, nationality, number of dependents.
2. Job workload:
  - Number of hospital on-call shifts per month (on-call shifts are 24 h long).
  - Number of days since last on-call shift.
  - Number of hours of direct patient interaction per day.
  - Number of hours at the hospital per day.
  - Out-of-hospital work: number of hours per day dedicated to study, preparation of clinical sessions, courses, or scientific writing outside of work.
3. Subjective work experience:
  - Perceived responsibility for the tasks.
  - Perceived difficulty of the tasks.
  - Perceived support from supervisors.
  - Perceived support from peers.

In the online survey, questions were administered in the following order: demographic characteristics, workload, subjective work experience, MBI, and ERQ.

### 2.4. Data Analysis

First, we conducted a descriptive analysis to report burnout levels in the sample. Next, a multivariable regression model was fit to explain the variance of each burnout subscale. Demographic variables, workload data, and subjective work experience were used as covariates in the first block. Then, the two emotion regulation strategies, namely emotional suppression and cognitive reevaluation, were entered in a second block to quantify the amount of variance of burnout that was explained by the emotion regulation strategies after the covariates were taken into consideration. Finally, an interaction between the covariates and emotion regulation strategies was added in a third block. This last step was included to explore whether the relationship between emotion regulation strategies and burnout differed as a function of scores in the covariates (i.e., moderation). We used a stepwise multiple regression procedure for each of the three burnout subscales. Here, we will only report the final models, including the relevant predictors only for clarity. 

## 3. Results

Residents’ levels of burnout are reported in Table 2. Approximately two-thirds of the sample presented high burnout symptoms.

Bivariate associations between emotion regulation strategies and burnout, along with the internal consistency of scales, are shown in Table 3. The use of emotional suppression strategies correlated with depersonalization (*r* = 0.22; *p* < 0.05), while the use of cognitive reevaluation strategies was associated with higher feelings of personal accomplishment (*r* = 0.35; *p* < 0.001). All scales showed good internal consistency.

### Prediction of Burnout Subscales

The multivariate regressions predicting burnout facets can be found in Table 4, Table 5 and Table 6. With regard to emotional exhaustion (Table 4), participants reported significantly lower levels of exhaustion when they felt supported by their supervisors, when they treated patients during more hours a day, and when they perceived less difficulty in their job. This model explained 21.7% of the variance in fatigue levels. None of the burnout variables explained the additional variance of emotional exhaustion after work conditions were taken into consideration.

Regarding depersonalization, residents reported lower levels when they devoted more hours to treating patients (Table 5). This association was stronger when they reported higher levels of cognitive reevaluation (we found a positive interaction between the use of cognitive reevaluation and the number of hours treating patients). Participants also indicated lower levels of depersonalization when they perceived more support from their supervisors. On the other hand, higher levels of depersonalization were associated with higher levels of emotional suppression. This model explained 22.3% of the variance of depersonalization.

The personal accomplishment subscale was positively associated with cognitive reevaluation. Interestingly, this association was weaker when residents perceived higher levels of supervisors’ support. The relation was also weaker in participants with higher scores of emotional suppression. We also found an interaction between emotional suppression and supervisors’ support in the prediction of personal accomplishment. Since the coefficients for both predictors were negative (non-significant and not reported in Table 6), this interaction means that the negative relationship between emotional suppression strategies and personal accomplishment is stronger when residents perceive that they receive more support from their supervisors. This model explained 34.8% of the variance of personal accomplishment.

## 4. Discussion

According to previous studies, physician burnout estimates in residents are generally high and up to 70% [41,46]. We found similar levels of burnout in our sample, indicating that many residents struggle with negative emotions during this particular training period. Overall, our results revealed that emotion regulation strategies were associated with residents’ burnout, which is in line with past research showing that emotional management is fundamental in healthcare professionals [17,47,48]. Specifically, emotional suppression was associated with depersonalization, and cognitive reevaluation was related to personal accomplishment. These associations occurred in the expected direction; that is, emotional suppression was associated with more burnout, while cognitive reevaluation strategies correlated with lower levels of this syndrome. Most importantly, for the present study, the contribution of both strategies remained significant when demographic variables and work-related factors were accounted for in a multivariate regression model.

Emotional suppression is an emotion regulation strategy characterized by inhibiting true emotions in favor of feelings that are required in a given situation [14]. However, this is psychologically costly, as the underlying appraisals of the situation are not being changed. Previous research in organizational settings has already evidenced that emotional suppression strategies are associated with increased levels of burnout [49]. Results in the present study indicate that this might also be the case in hospital residents. Interestingly, though, our analyses suggested that this emotion regulation strategy might contribute mostly to depersonalization. The depersonalization scale measures an individual’s tendency to be cold and unfeeling towards people they care for, provide service to, or instruct at work [41]. What our results suggest is that hiding one’s true undesired feelings (e.g., fear of failure, insecurity) as a protective strategy may increase the emotional distance between residents and patients. Because healthcare professionals’ empathy is known to predict beneficial outcomes in the patient [50], we interpret our results as indicating that residents’ use of emotional suppression as an emotion regulation strategy may be maladaptive due to its positive association with depersonalization. 

As opposed to emotional suppression, our results support the idea that cognitive reevaluation may be an adaptive emotion regulation strategy for hospital residents. Cognitive reevaluation involves reevaluating a situation to give it a new meaning, thus changing the emotional response it produces. Even though it may require a higher initial effort to find alternative interpretations, it seems to be a better strategy in the long term. Past studies exploring the relationship between cognitive reevaluation and personal outcomes were not conclusive [49]. However, theory on emotion regulation states that cognitive reevaluation strategies are preferred over emotional suppression efforts because only the former leads to a change in experienced emotion [14]. Our results support this hypothesis. Specifically, what our analyses indicate is that residents using cognitive reevaluation strategies tend to present increased levels of personal accomplishment. In our opinion, it is possible that the ability to reappraise a situation in order to change one’s feelings results in a more successful adaptation to the environment. In the case of hospital residents, this appears to facilitate a feeling of personal achievement at work. Note, though, that the correlational nature of our data prevents us from drawing causal conclusions from our results.

An interesting finding from the present study is that the relationship between certain predictors of burnout (i.e., emotion regulation strategies and work-related factors) and burnout might be more complex than we thought. For example, the number of hours spent with the patient might be associated with reduced depersonalization, but they appear to synergize with cognitive reevaluation strategies: residents with higher levels of cognitive reevaluation seem to benefit more from spending more hours in direct patient care. The specific number of patients attended was not assessed. Conversely, we focused on the time spent with the patients. Perhaps less time spent with patients could be associated with greater time constraints, which could explain the reduced depersonalization levels in those who dedicate more time to patients.

Our analyses also indicated that the relationship between emotion suppression strategies and burnout might be modulated by work-related factors. Specifically, while cognitive reevaluation strategies were associated with increased levels of personal accomplishment, the strength of this association was increased when residents perceived low levels of support from their supervisors. Studies frequently indicate that having a supportive supervisor is associated with better outcomes in the employee [51,52]. Together with existing research, our results revealed that having a supportive supervisor may also reduce the need for the use of emotion regulation strategies, namely cognitive reevaluation. The use of this emotion regulation strategy may be more important for residents’ burnout when supervisors are not supportive. 

Both socio-demographic factors and work conditions have not been consistently associated with residents’ burnout [9]. For instance, race/ethnicity, primary language, and cultural background have been found to be associated with burnout levels in residents in some studies [53,54], but not in others [55,56]. Similarly, some studies have indicated an association between burnout and some work conditions, such as working hours and number of on-call nights per week [57], but others have not [55,56], and implementing a reduction in the number of work hours has not been shown to reduce burnout [58]. Our results might suggest an explanation for these inconsistencies. Specifically, it is possible that the contribution of these variables to burnout levels is moderated by the residents’ emotion regulation style.

The present study certainly has limitations. First, the fact that the number of nurse residents was importantly lower than that of medical residents limits the generalizability of the results to a nursing population. However, burnout problems in nurses and physicians tend to be similar [59], so it is possible that the present study findings might be useful for both professional groups. Similar to other single-center studies, one of the limitations of the present study’s findings is that the results may not be generalizable to other contexts with a different local culture or organizational settings. Our results would need to be confirmed in different contexts. Another limitation of the current investigation is that the statistical approach used (stepwise multivariate regression) is bottom-up, data-driven, and not theoretically driven. This method was selected because of the exploratory nature of the study (no clear hypothesis about the interactions) and to obtain a more parsimonious model with a more manageable number of variables. Furthermore, causal attributions cannot be drawn because of the cross-sectional nature of the study. We want to note that supervisor support, which was a relevant predictor in our models, can be confounded with other variables, such as residents’ work histories [60]. Future research should (a) identify the distinct effect of these two variables; (b) explore the influence of psychological and situational variables not included in our work, such as personality traits, the type of assigned tasks/workplaces, and the number of patients cared for; and (c) examine the moderating effects of other demographic variables, such as age and sex/gender.

## 5. Conclusions

Despite the aforementioned study shortcomings, our results may have important research and clinical implications. Research-wise, it showed that work-related and emotion regulation variables should be considered together when explaining burnout, as both can contribute unique variance to the prediction of this syndrome. Moreover, it revealed that burnout is indeed a complex construct that requires interaction models between psychological and work conditions. Clinical implications can also be drawn from this study. A meta-analysis concluded that the effectiveness of existing treatments for job burnout in mental health providers, especially those that are organization-directed, is very limited [51]. The study also indicated that a broader range of interventions, especially person-directed, is needed. We believe that our results align with these conclusions and support the inclusion of a novel person-directed target, namely emotion regulation, in intervention programs aiming to reduce residents’ burnout. Importantly, our study findings also support the idea that treatment should be personalized to maximize its effectiveness. For example, it is possible that increasing supervisor support and decreasing the use of emotion suppression is most beneficial to increase depersonalization problems, while tackling cognitive reevaluation might be preferred for individuals with deficits in personal accomplishment. Similarly, the moderation analyses revealed that certain initiatives (i.e., reducing the number of hours of patient care) might not be recommendable for all individuals (i.e., residents with high levels of cognitive reevaluation who spent more hours with the patient had less burnout), which might also be important for personalized interventions.

## Figures and Tables

**Table 1:** Demographic characteristics of the sample.

*n* = 105	%
Sex (Women)	68.6
Marital Status	
Single	88.6
Married	11.4
Nationality	
Spanish	84.9
Latin American	10.3
Other European	4.7
Specialty	
Medicine	62.9
Surgery	16.2
Laboratory	7.5
Nursing	14.5
Year of Residency	
First	30.5
Second	41.0
Third	22.9
Fourth	5.7

**Table 2:** Distribution of burnout in the sample.

*n* = 105	Fatigue	Depersonalization	Personal Fulfillment
High	62.9%	68.6%	67.6%
Medium	25.7%	26.7%	22.9%
Low	11.4%	4.8%	9.5%

**Table 3:** Psychometric properties and Pearson correlations between burnout and cognitive strategies.

	Psychometric Properties			Pearson Correlations				
*n* = 95	Cronbach’s *α*	Mean	*SD*	1	2	3	4	5
Burnout								
1. Emotional exhaustion	0.80	29.8	9.0					
2. Depersonalization	0.88	12.7	5.3	0.56 ***				
3. Personal accomplishment	0.82	41.1	5.3	−0.24 *	−0.15			
4. Burnout full scale				0.89 ***	0.75 ***	−0.55 ***		
Emotion regulation strategies								
5. Suppression	0.81	13.1	5.7	0.15	0.22 *	−0.04	0.18	
6. Reevaluation	0.73	28.3	6.9	0.11	−0.06	0.35 ***	−0.08	0.03

Note: 10 residents did not complete the whole assessment protocol, so analyses could not be performed. * *p* < 0.05; *** *p* < 0.001.

**Table 4:** Linear regression model results for the emotional exhaustion subscale.

*n* = 88	B (Unstandardized)	CI (95%)	Std. Err	B (Standardized)	*t*
Supervisor support	−3.00	−4.85, −1.15	0.93	−0.32	−3.23 **
Perceived difficulty	4.46	2.21, 6.70	1.13	0.38	3.94 ***
Daily hours of patient care	−0.76	−1.40, −0.11	0.32	−0.23	−2.34 *

* *p* < 0.05; ** *p* < 0.01; *** *p* < 0.001.

**Table 5:** Linear regression model results for the depersonalization subscale.

*n* = 88	B (Unstandardized)	CI (95%)	Std. Err	B (Standardized)	*t*
Daily hours of patient care	−0.69	−1.08, −0.30	0.20	−0.36	−3.53 ***
Supervisor support	−1.36	−2.44, −0.28	0.54	−0.25	−2.51*
Suppression	1.12	0.05, 2.19	0.54	0.20	2.07 *
Reevaluation * Daily hours of patient care	1.31	0.15, 2.48	0.59	0.22	2.24 *

* *p* < 0.05; *** *p* < 0.001.

**Table 6:** Linear regression results for the personal accomplishment subscale.

*n* = 88	B (Unstandardized)	CI (95%)	Std. Err	B (Standardized)	*t*
Reevaluation	1.87	0.76, 2.97	0.55	0.35	3.37 **
Suppression *Reevaluation	−1.68	−2.57, −0.78	0.45	−0.35	−3.72 ***
Reevaluation *Supervisor support	−1.31	−2.16, −0.46	0.43	−0.30	−3.08 **
Suppression *Supervisor support	1.36	0.16, 2.56	0.60	0.24	2.25 *

* *p* < 0.05; ** *p* < 0.01; *** *p* < 0.001.

## Supplementary Materials

The following are available online at https://www.mdpi.com/1660-4601/17/21/7816/s1, Table S1. Subjective Work Experience items, Table S2. Job Workload items.
