# Supplementary material for: Emotion Regulation Strategies, Workload Conditions, and Burnout in Healthcare Residents
Source: Int J Environ Res Public Health. 2020 Oct 26;17(21):7816. doi: 10.3390/ijerph17217816 (PMC7663662; doi:10.3390/ijerph17217816)
Supplement: Supplementary file 1 [file ijerph-17-07816-s001.pdf]

Supplementary file

Table S1. Subjective Work Experience items

	Very Low	Low	Average	Hig h	Very High
Realice una valoración subjetiva de su Responsabilidad laboral/ Make a subjective assessment of your Labor Responsibility					
Realice una valoración subjetiva de su dificultad laboral/Make a subjective assessment of your job difficulty					
Realice una valoración subjetiva del apoyo recibido por sus supervisores/Make a subjective assessment of the support received by your supervisors					
Realice una valoración subjetiva del apoyo recibido por sus compañeros/Make a subjective assessment of the support received by your colleagues					

Table S2. Job Workload items

¿Cuántas horas de guardias al mes?/ Number of hospital on-call shifts per month (on-call shifts are 24h long).	
¿Cuantos días hace desde su última guardia, horas extra o atención continuada? / How many days has it been since your last on callhift, overtime, or continuous care?	
Estime el número de horas que dedica a atender pacientes en una jornada laboral ordinaria./ Estimate the numberof hours of direct patient interaction per day	
Estime la media de horas al día de su horario laboral, sin incluir guardias, en su centro de trabajo (incluyendo: atención continuada, quirófanos de tarde, sesiones clínicas...) / Estimate the average number of hours at the hospital per day not	

including shifts, in your workplace (including: continuous care, afternoon operating rooms, clinical sessions ...)	
Estime la media de horas al día fuera de su horario laboral que dedica a su trabajo (horas de estudio, conferencias, cursos, preparación artículos, ...) / Estimate the average number of hours per day dedicated to study, preparation of clinical sessions, courses, or scientific writing outside of work.	
